# Special Issue: “Viral Genetic Diversity”

**DOI:** 10.3390/v14030570

**Published:** 2022-03-10

**Authors:** Jean-Michel Heraud, Anne Lavergne, Richard Njouom

**Affiliations:** 1 National Reference Laboratory for Rabies and Viral Encephalitis, Institut Pasteur de Dakar, Dakar 12900, Senegal; 2 Laboratoire des Interactions Virus-Hôtes, Institut Pasteur de la Guyane, Cayenne 97306, French Guiana; alavergne@pasteur-cayenne.fr; 3 Virology Department, Centre Pasteur du Cameroun, Yaounde, Cameroon; njouom@pasteur-yaounde.org

Some say that small is beautiful, and if beauty could be measured by levels of diversity and complexity, we could definitely say that viruses are beautiful. This fascinating particle of life has developed complex mechanisms to infect a large variety of eukaryote and prokaryote hosts. Although some mechanisms such as the exchange and duplication of genes can occur, genetic diversity amongst viruses is mainly generated through inter- and intra-species genetic recombination and mutation. Nucleotide substitution is the most common mechanism of genetic variation and results from errors during the replication of the virus by viral polymerases. The lack of proofreading and repair activities by RNA virus polymerases explains why RNA viruses have higher mutation frequencies than DNA viruses. Nevertheless, some DNA viruses can avoid high-fidelity replication mechanisms and have high mutation frequencies [1,2,3]. The inherent genetic variability of viruses has consequences in terms of epidemiology, as it allows viruses to adapt to new hosts and escape the host’s immune pressures. Thus, genetic variation may impact diagnosis performance, antiviral treatment efficacy and vaccine development.

In this Special Issue of *Viruses*, we collected peer-reviewed research articles focusing on different aspects of viral genetic diversity.

Interestingly, Armero et al. suggest human-to-human transmission of nonsynonymous intra-host variants of SARS-CoV-2 [4]. They propose a generalized use of next-generation sequencing (NGS) data to better decipher transmission-impacting factors and the evolution dynamic of quasispecies of SARS-CoV-2 over time. The NGS approach has proven its ability to quickly identify variants of concern (VOCs) that are responsible for different waves. During this SARS-CoV-2 pandemic, whole-genome studies available on the GISAID (Global Initiative on Sharing Avian Influenza Data) database have also changed some paradigms regarding coronavirus evolution. Indeed, since these RNA viruses have a proofreading polymerase mechanism that regulates fidelity and diversity [5], it was expected that SARS-CoV-2 would evolve slowly enough to allow vaccines to confer long-lasting protection. The recent emergence of variants of concern (VOCs) seems to be driven by an acceleration in the SARS-CoV-2 mutation rate [6], which could impact the duration of the protective immunity provided by vaccines. In the current issue, the structure-function of recent variants is analyzed, and implications regarding clinical, diagnostic, therapeutic and public health are presented by Singh et al. [7].

Another respiratory virus, the respiratory syncytial virus (RSV), poses a public health problem in terms of morbidity and mortality. The annual burden of the disease associated with RSV infection is high, particularly in countries with low resources and where access to care is limited, resulting in elevated mortality among children under five years of age [8]. To reduce the burden of RSV-associated disease, it is important to develop new vaccines and antiviral medicines; however, for this, it is important that countries survey this virus in order to monitor its spread and molecular evolution. In this Special Issue, Lee et al. explore the genetic diversity of RSV in Taiwan, which could contribute to a better understanding the epidemiology of RSV, as well as better predictions of its evolution over time at the local level [9].

Africa and South America encompass hotspots of biodiversity, not only among their fauna and flora, but also all along their living landscapes. Somehow, the diversity of species impacts the diversity of viruses. We discovered, in this Special Issue, that viral diversity in the neotropical rodents of the Amazonian basin is influenced by their habitat types [10]. In particular, the richness of viruses is higher in undisturbed environments than in peri-urban areas. This richness of viruses is also observed in birds from Africa, and Luo et al. describe bird-related rhabdoviruses, considered as new species of the genus Sunrhavirus [11]. Interestingly, the authors raise the question of whether bird-related rhabdoviruses could be an arbovirus that infects birds, considering the close genetic relationship of these rhabdoviruses with other insect-related sunrhaviruses. The work conducted by Mamimandjiami et al. offers new insights into the epidemiology of Human Herpes Virus type 8 (HHV-8) and its genetic diversity in the central African Country of Gabon [12].

Another example of the high degree of viral genetic diversity is in Picobirnaviruses (PBVs). These small, segmented RNA viruses have been detected in a wide variety of hosts, both vertebrate and invertebrate, as well as in the environment. Perez et al. have published their work in this issue, which shows that the optimization of phylogenetic analysis is essential to better understand the phylogenetic diversity of PBVs [13]. Following their work, the authors propose a new nomenclature to indicate the species of each segment of PBV.

In this Special Issue, we sought to highlight recent advances in understanding the genetic diversity of viruses. We are grateful for the tremendous work conducted by the authors of the articles published. The diversity of topics presented in these papers reflects the diversity of virology, and we are convinced that our issue will provide interesting new knowledge and insight to the virology community.
